# Opening the window to the children’s mind: the superior efficacy of open-ended physical games in the development of attention and socio-emotional skills

**DOI:** 10.3389/fpsyg.2025.1511559

**Published:** 2025-02-19

**Authors:** Shanshan Lyu, Weixiao Zhang

**Affiliations:** ^1^School of Physical Education, China University of Mining and Technology, Xuzhou, China; ^2^Centre for Postgraduate Studies, Perdana University, Kuala Lumpur, Malaysia; ^3^Business School, University of Technology Sydney, Sydney, NSW, Australia

**Keywords:** open exercise, closed exercise, attention, socio-emotional skills, physical games, children

## Abstract

**Background:**

The promotional effects of physical exercise on children’s attention and social–emotional skills have been widely confirmed. However, the advantages of open physical games in intervention effects still need further exploration. Therefore, this study discusses the intervention effects of open physical games on children’s attention and social–emotional skills.

**Methods:**

This study adopted a quasi-experimental design. Two administrative classes were divided into the experimental group (15 boys and 16 girls) and the control group (16 boys and 15 girls) using the coin-toss method. The intervention intensity was moderate (60–69% of HRmax), lasting for 12 weeks, with 3 sessions per week, each lasting 40 min. Both groups focused on the teaching of fundamental motor skills (FMS). The experimental group was intervened with open physical games, while the control group was intervened with closed physical games. The Adolescent Attention Test was used to measure attention quality; the Children’s Social and Emotional Skills Scale was used to measure social–emotional skills; the Test of Gross Motor Development-Third Edition was used to measure locomotors and manipulative skills; and the Fundamental Movement Skill Development Test for Children Aged 3 to 10 was used to measure stability skills. Based on SPSS 25.0 and GraphPad Prism 8 software, statistical analysis was conducted using independent samples *t*-tests, χ^2^ tests, MANOVA, and Pearson partial correlation analysis.

**Results:**

After the intervention, open physical games had a more positive effect on children’s attention distribution (*F* = 6.223, *p* = 0.022, *η*^2^ = 0.090). Open physical games had a more positive effect on children’s self-awareness (*F* = 11.027, *p* = 0.002, *η*^2^ = 0.165), others’ awareness (*F* = 10.315, *p* = 0.002, η^2^ = 0.156), collective awareness (*F* = 6.494, *p* = 0.014, *η*^2^ = 0.104), collective management (*F* = 12.108, *p* = 0.001, *η*^2^ = 0.178), and overall social–emotional skills (*F* = 38.453, *p* < 0.001, *η*^2^ = 0.407). Open physical games had a more positive effect on locomotors skills (*F* = 6.458, *p* = 0.014, *η*^2^ = 0.104), manipulative skills (*F* = 6.380, *p* = 0.014, *η*^2^ = 0.102), and overall FMS (*F* = 10.333, *p* = 0.002, *η*^2^ = 0.156). In addition, there is a certain degree of positive correlation between FMS, attention, and social–emotional skills (*p* < 0.05).

**Conclusion:**

Open physical games have superior effects on children’s attention, social–emotional skills, and FMS compared to closed physical games. This has guiding significance for subsequent physical education practices and the formulation of public health policies.

## 1 Introduction

Attention and social–emotional skills are key competencies that urgently need to be developed during childhood. Attention refers to the selective cognitive process of effectively balancing and processing information when facing multiple tasks and goals (Yantis, 2000). Children need to participate in attention as they recognize, adapt to, and transform the world. The learning process and thinking development of children rely on attention. A study (Sigman et al., 1986) suggests that attention is a necessary condition for the brain to receive external information stimuli and transmit them to the memory system, and it determines the intellectual development to a certain extent. Therefore, numerous studies (Deutsch et al., 2008; Harris et al., 2005) have found that attention is closely related to children’s intellectual development and academic performance. Children with extraordinary intelligence have a higher level of attention and higher learning efficiency (Merrienboer et al., 2002). In addition, the development of attention helps children learn to regulate emotions (Wadlinger and Isaacowitz, 2011), which is crucial for dealing with interpersonal relationships. Social–emotional skills refer to an individual’s abilities to recognize, express, and regulate emotions, as well as to establish and maintain relationships with others during social interactions (Abrahams et al., 2019; Malti and Noam, 2016). The learning and development of these skills typically begin in early childhood and are gradually formed through education and practice in the family, school, and community. These skills play an important role in preventing problematic behaviors, improving academic performance, promoting mental health, and establishing successful interpersonal relationships (Ashdown and Bernard, 2012).

Inattention in class has already become a common problem among Chinese children. According to research (Yin, 2023; Zhao and Guan, 2020), nearly 80% of students will be distracted in class, and approximately 50% ~ 80% of children will continue to have problems with inattention in class until adolescence, while around 40% of them will still have such problems until adulthood. In addition, childhood is a stage of rapid psychological development of social–emotional skills, especially as children transition from simple family and kindergarten environments to complex school environments. They will face various sources of stress such as academic demands, peer relationships, and teacher-student relationships. If they cannot use positive social–emotional skills to cope with the above stress sources, it would be very detrimental to the children’s development. Yu et al. (2014) found that the detection rate of social difficulties among Chinese children is about 15%. Due to the lack of social skills, these children may not be able to effectively express their needs and emotions, leading to difficulties in social interactions, and thus encountering challenges in establishing and maintaining interpersonal relationships. Therefore, it is urgent to improve children’s attention and social–emotional skills.

Physical exercise is an important means of improving attention and enhancing social adaptability (Kalina et al., 2016; Vhavle et al., 2019). Spitzer and Hollmann (2013) found that even jogging can improve children’s attention and positive social behavior. Vanhelst et al. (2016) discovered that exercising for one hour a day can significantly improve cognitive indicators such as attention, executive function, and working memory. Reloba-Martínez et al. (2017) found that high-intensity exercise has a significant beneficial impact on attention span and selective attention. Physical exercise can promote the increase in the volume of the hippocampus in the brain, and an increase in hippocampal volume implies that attention functions may be enhanced (Aly and Turk-Browne, 2017; Triviño-Paredes et al., 2016). In addition, physical exercise can promote the development of the prefrontal cortex of the brain, and the prefrontal cortex has a regulatory role in attention control (Choi et al., 2015). Through physical exercise, the neural connections in this area can be optimized, and the transmission of information between neurons becomes more efficient. As a result, children can better inhibit the interference of irrelevant information and focus their attention on important tasks (Chang et al., 2015; Choi et al., 2015). There is a causal relationship between physical exercise and social and emotional skills as well as social adaptability (Ikenouchi-Sugita et al., 2013). Physical activities that combine teamwork, competition, and social interaction can enhance social opportunities and individual self-confidence, thereby cultivating the team spirit and cooperative awareness of adolescents. Physical exercise can also enhance the development of self-control abilities, which in turn promotes the development of social emotional skills (Shi and Feng, 2022). In addition, physical exercise promotes the release of hormones such as endorphins, thereby improving mood, reducing anxiety, and enhancing mental health (Dinas et al., 2011). The improvement of mental health can further promote the development of social emotional skills (Colomeischi et al., 2022). For example, by enhancing the ability to regulate emotions, it helps individuals manage their emotions better, thereby enabling them to interact more comfortably in social situations. In summary, physical exercise has a positive promotional effect on improving attention and enhancing social emotional skills.

With the continuous progress of research, researchers have gradually paid attention to the effects of different types of exercise on attention and social and emotional skills. Shi J. et al. (2022), Shi P. et al. (2022), and Shi et al. (2024) categorized exercise types into open and closed exercises based on the unpredictability of the exercise environment. Open exercise refers to the skill of performing movement tasks in an unpredictable environment; closed exercise refers to the skill of performing movement tasks in a stable, predictable environment (Shi J. et al., 2022; Shi P. et al., 2022; Shi et al., 2024). Petrosini et al. (2009) suggest that the unpredictability of the environment provides more cognitive resources, which is conducive to promoting cognitive development. Therefore, many studies (Shi J. et al., 2022; Shi P. et al., 2022; Shi et al., 2024) have conducted research on open and closed skills divided by the unpredictability of the exercise environment. Tsai et al. (2016) found differences in visual–spatial attention between groups who participated in open exercises (such as badminton, table tennis, etc.) and closed exercises (such as jogging and swimming, etc.) over the past 2 years. The results showed that both open and closed exercises had shorter reaction times compared to the control group, with the potential benefits of open exercises being even better. A narrative review by Shi and Feng (2022) points out that the effect of open exercise on children’s cognitive and social–emotional skills is superior to that of closed exercise, mainly because the former is accompanied by rich environmental stimuli and interpersonal interaction elements. Open exercises often take place in environments without fixed rules, where children naturally improve the flexibility of their attention by adjusting their movements according to the scene and rules during the process of free exploration and trial. In addition, open exercises provide opportunities for social interaction, which can cultivate children’s social skills such as communication, negotiation, and cooperation with others (Shi and Feng, 2022). It can also help children better understand and manage their emotions during the exercise process, thereby enhancing their social–emotional skills.

However, there are some limitations in current studies. Firstly, the results of open exercise intervention on attention and socio-emotional skills are inconsistent. For example, Giglia et al. (2011) and Tsai et al. (2016) found that open exercise improves the effect on visual–spatial attention better than closed exercise, while Chueh et al. (2017) showed that both open and closed exercises have positive promoting effects on visual–spatial attention. In addition, many studies (Che, 2014; Wu, 2018; Yin, 2018) have found that collective ball game-dominated open exercises have better promoting effects on interpersonal adaptation and the quality of sports friendship than closed exercises. The main reason is that open exercise helps to enhance self-esteem, intimacy and exercise pleasure. However, Cao (2016) found that there are no significant differences in social adaptation and positive psychological qualities among college students of different physical exercise programs. Secondly, the benefits of open exercise in promoting attention and socio-emotional skills need further investigation. Attention includes four characteristics: attention span, attention stability, attention distribution, and attention transfer (Yin, 2003). However, previous studies have not been sufficiently thorough in examining the effects of exercise interventions on these dimensions, and it is still unclear what type of exercise is more beneficial for what kind of attention quality (De Sousa et al., 2019). Moreover, although social adaptation and socio-emotional skills are somewhat related, they are not entirely the same, so it is necessary to conduct specific research on open exercise interventions for socio-emotional skills. Finally, previous studies(Chang et al., 2015; Chueh et al., 2017; Vhavle et al., 2019; Wu, 2018; Yin, 2018) have often been based on specific exercise programs (such as basketball, table tennis, swimming, cycling, etc.) as intervention carriers to explore the effectiveness of different programs in intervening attention and social–emotional skills. Children engage more in complex game activities based on motor skill learning during their childhood, and the promoting benefits of physical games remain to be explored.

Based on these studies, this study examined the benefits of open physical games in promoting attention and socio-emotional skills in children by using both open and closed physical games as instructional interventions. Through this study, on one hand, it is expected to promote the development of exercise intervention research on attention and socio-emotional skills, enriching the related exercise intervention methods. On the other hand, it is hoped to encourage policymakers and school physical education workers to pay attention to the positive benefits of open exercise, and promote practitioners to adopt open exercise to enrich the environment with stimuli, thereby enhancing children’s cognitive and social adaptation abilities.

## 2 Methods

### 2.1 Participants

This study used G*Power 3.1 software to calculate the sample size, referring to the study by De Greeff et al. (2018), with an effect size of 0.43, a significance level of 0.05, and a power of 0.95. The calculation indicated that a total of 60 participants were needed for both groups. Considering a 10% possibility of attrition, the study planned to recruit a total of 66 participants. Considering the convenience of study, this study selected an elementary school in Xuzhou City, Jiangsu Province, China, as the research site. The study obtained informed consent from the school principal and chose two natural classes of the first grade, with a total of 70 students, to conduct the experiment. All parents of the children participating in the experiment have signed the informed consent form. The two classes were randomly divided into an experimental group and a control group using the coin toss method, with both groups consisting of children aged between 7 to 8 years old. The experimental group included 17 boys and 18 girls, while the control group included 18 boys and 17 girls. During the experiment, 1 boy in the experimental group missed more than one-third of the classes, and 1 boy and 2 girls in the experimental group and 2 boys and 2 girls in the control group provided non-standard answers to the questionnaires. Therefore, the data from these participants were excluded. Ultimately, 31 participants in the experimental group (15 boys and 16 girls) and the control group (16 boys and 15 girls) were included in the analysis. This study was approved by the Scientific Research Ethics Committee of China University of Mining and Technology.

### 2.2 Experimental design

This study used cluster random assignment to divide the two classes into the experimental class and the control class. This study assigned the two classes the codes Class 1 and Class 2. One researcher randomly assigned the two classes to the experimental and control groups by flipping a coin. If the coin landed heads up, Class 1 was designated as the experimental group, and Class 2 as the control group; if tails were up, Class 1 was the control group, and Class 2 was the experimental group. This study employs a quasi-experimental design for the experiment, where the experimental group children engage in open-ended exercises guided by physical games, while the control group children learn through closed physical games. The study invited two physical education teachers to implement the teaching plans for both the experimental and control groups, to minimize the potential cross-influence of a fixed teacher teaching different classes. Moreover, the two physical education teachers have the same professional titles and years of teaching experience, ensuring that the factor of teacher experience will not interfere with the experimental results. During the experiment, all teaching materials, experimental procedures, and scheduling arrangements were kept highly consistent. We provided the students of both classes with exactly the same learning materials and strictly followed the predetermined timetable to carry out various experimental activities, in order to avoid interference with the experimental results due to factors such as teaching content and time. Additionally, the study adopted a single-blind strategy for the participants to effectively avoid the Hawthorne effect among students. During the testing of attention, social–emotional skills, and fundamental movement skills (FMS), the testers were unaware of the students’ basic information and did not know which class the students belonged to.

### 2.3 Teaching program

According to the requirements of the *Compulsory Education Physical Education and Health Curriculum Standards* in China, the learning content of physical education classes for first-grade primary school students is the practice of FMS (Ministry of Education of the People's Republic of China, 2022). Therefore, the main teaching content for both the experimental group and the control group is FMS. The FMS are fundamental movement learning patterns that do not naturally occur in the human body and are the foundation for complex motor skills and physical activities (Barnett et al., 2016; Fisher et al., 2005). The FMS typically includes three categories: stability skills, locomotors skills, and manipulative skills (Barnett et al., 2016; Fisher et al., 2005). Stability skills refer to the abilities that involve maintaining body balance and stability, such as standing, sitting, and walking. Locomotors skills refer to the abilities that involve moving the body from one place to another, such as running, jumping, sliding, and crawling. Manipulative skills refer to the abilities that involve using the hands or feet to control or manipulate objects, such as throwing, catching, kicking, and striking. The FMS is the foundation for physical activity and specific sports skills, and it is crucial for the physical and mental health development of children and adolescents (Barnett et al., 2016; Stodden et al., 2008).

The teaching experiment was conducted in the indoor gymnasium of the intervention school. According to the study by De Sousa et al. (2019), both acute and long-term exercise are beneficial for the improvement of children’s attention skills, and long-term exercise lasting more than 8 weeks has a more positive effect. Therefore, this study, in combination with the class schedule of the intervention school, set the intervention period to 12 weeks, with 3 classes per week, and each class lasting for 40 min. The first and second classes of the first week, as well as the second and third classes of the twelfth week, were designated as test weeks. Therefore, a total of 32 classes were conducted, with a total intervention time of 1,280 min. The time arrangement for each class is as follows: the warm-up and cool-down time is approximately 10 min each, and the basic motor skills practice time is about 30 min. It should be noted that this time arrangement is not set in stone but is adjusted appropriately according to the actual situation.

This study, based on the actual situation of the students and following the principle of gradual progression, reasonably arranged the content of each class’s exercises, totaling 6 stability skill training classes, 10 locomotors skill training classes, 10 manipulative skill training classes, and 6 composite skill training classes. The stability skill training activities include stretching, pushing and pulling, twisting, hanging, supporting and pushing and pulling, and balancing, etc. The locomotors skill training activities include heel walking, high and low man walking, horse-step running, chasing running, pad running, running and jumping steps, dodging, crawling, etc. The manipulative skill training activities include various throwing, passing, striking, kicking, catching, and dribbling activities. In addition, the composite skill training mainly involves guiding students to participate in a variety of activities, such as practicing changing direction, path, and rhythm while dribbling, passing and receiving small soccer and basketball during various movements, running across various obstacles, and making different stopping actions in chasing running according to different signals. Through composite exercises, students’ sports experience can be enriched, and students’ perception of changes in time and body can be improved.

For the experimental group, this study employed physical games with a more unpredictable environment for teaching. Open-ended physical games provide a rich physical environment and more interpersonal interaction processes (Shi and Feng, 2022), which can enhance children’s perception of their body’s position and movement in space, as well as their perception of the surrounding environment. This helps children flexibly switch their attention processes during the game. In addition, group-oriented open-ended physical games are also an important means of improving children’s social adaptation, so this type of game is emphasized in this study. For example, traditional Chinese games such as “Eagle Catches Chicken” and various chasing and dodging games are used to improve students’ mobility skills. Obstacle chase games are used to enhance students’ locomotors skills. Various obstacles, such as low hurdles and barriers, are set up in the field. During the chase run, students not only have to cross these obstacles, but also perform stop actions according to different signals issued by the coach, such as standing on one foot or squatting. Additionally, games like “Throwing and Catching Beanbags” and “Table Tennis Ball Carrying Competition” are employed to enhance students’ manipulative skills. The game “Ball Battle Royale” is used to enhance students’ manipulative skills. Students are divided into several groups. During the ball - handling process, each group of students needs to make changes in direction, path, and rhythm according to the coach’s instructions, while also avoiding interceptions from other groups. For the control group, closed physical games with fixed environmental backgrounds and low variability are used for teaching. For example, in the “Balance Beam Challenge” game, students are required to start from the starting point and pass through the balance beam to the finish line at the slowest and most stable speed possible within a specified time (e.g., 30 s), during which the environmental background is predictable. Additionally, the scenarios of games such as fixed-distance dribbling shuttle run and ball - juggling count within a limited area are all fixed. Previous studies (Chen et al., 2014; Xiong et al., 2018) have suggested that moderate-intensity exercise (60–69% of maximum heart rate) has a better promoting effect on children’s cognition. Therefore, this study used heart rate monitors to monitor the students’ heart rates during each class to ensure that the heart rate was maintained at this intensity.

### 2.4 Variables and tools

#### 2.4.1 Attention

The adolescent attention test compiled by Yin (2003) from Beijing Normal University in 2003 mainly tests the distribution, span, stability and transfer of attention. Subsequent studies (Ni, 2018; Zhou et al., 2023) have migrated this test to the children’s group. After a rigorous standardization process, it has high reliability and validity, and can accurately assess children of different ages and backgrounds. The attention test is consistent with the results of self-evaluation by children themselves, as well as evaluations by parents and teachers, thus it has high validity (Yin, 2003). The test includes four sub-tests: attention distribution, attention span, attention stability, and attention transfer. The attention distribution of children is tested using a shape discrimination test, where the test items consist of two rings of different sizes with notches. Due to the different directions of the notches, many similar but different shapes are formed. Participants are required to identify the two specified shapes and mark them with a “√.” The attention span of children is tested using a select four circles test, where the test items are small squares with different numbers of circles drawn in them. Participants are required to find the squares with four circles and mark them with a “√.” The attention stability of children is tested using a visual tracking test, where the test items are several curves starting from the left and ending on the right. The test includes two sets, A and B, with set A containing 10 curves and set B containing 25 curves, totaling 35 curves. Participants are required to track a curve with their eyes starting from the left and write the sequence number of the curve at the start in the square at the end of the curve on the right. The attention transfer of children is tested using an addition and subtraction test, where the test items are calculations with natural numbers from 1 to 9. Participants are required to alternate between addition and subtraction operations and write the result between the two numbers. In the above four tests, each correct answer is worth 1 point, and the final number of correct answers is the participant’s final score in that test. The higher the score in each dimension, the better the corresponding attention quality. In addition, the test time for attention distribution, attention span, and attention transfer is 3 min, while the test time for attention stability is 2 min, and the interval between different attention quality tests is 2 min.

#### 2.4.2 Socio-emotional skills

This study utilizes the Children’s Social and Emotional Skills Scale developed by the Social Emotional Learning and School Management Improvement Project team from the Ministry of Education of China and the United Nations International Children’s Emergency Fund to assess children’s social and emotional skills (Shi J. et al., 2022; Shi P. et al., 2022; Zhou, 2023). The scale consists of 69 items across six dimensions: self-awareness, self-management, others’ awareness, others’ management, collective awareness, and collective management. Participants are required to evaluate each item using a four-point rating system under the guidance of their parents, where 1 to 4 represent completely not applicable, partially applicable, mostly applicable, and completely applicable, respectively. The higher the score a child receives, the higher their level of social and emotional skills is indicated.

Children aged 7 ~ 8 have already developed a certain level of self - awareness and expressive ability. At this age, children begin to gradually understand their own emotions, thoughts, and relationships with others. Having children conduct self - reports can directly obtain their inner feelings and experiences. Compared with evaluations by others, self-reports can more truly reflect children’s own cognition of social emotions. This study does not allow parents to dominate the children’s answers. Instead, assistance is provided when necessary, mainly to help children understand some more abstract words or expressions, ensuring that the children clearly understand the questions. In addition, to ensure the standardization of the “parental guidance” process, we provided parents with a detailed instruction manual before the project were implemented, clearly stating the principles and methods of guidance. Self-awareness item example: “When emotions are in extreme situations, one can be aware of them.” Self-management item example: “Possess a range of skills to deal with negative emotions, such as worry, anger, and anxiety.” Others’ awareness item example: “Knowing that the way one expresses emotions can affect how others feel.” Others’ management item example: “Being able to resolve conflicts with friends and family so that everyone is satisfied with the outcome.” Collective awareness item example: “Understanding what it feels like to belong to a group and how a sense of belonging can affect one’s emotions.” Collective management item example: “Being able to resolve conflicts at the group level through negotiation, compromise, and other methods.” The internal consistency coefficient of the scale is 0.972, and the internal consistency coefficients for each dimension are all above 0.8, indicating good reliability (Shi J. et al., 2022; Shi P. et al., 2022; Zhou, 2023).

#### 2.4.3 FMS

This study utilized the Test of Gross Motor Development-Third Edition (TGMD-3) developed by Ulrich (2013) to measure children’s locomotors and manipulative skills. Chinese scholars Li et al. (2022) validated the reliability and validity of TGMD-3 in assessing FMS among Chinese children aged 3–12 years. They found that the difficulty level of the 13 items of TGMD-3 ranged from 0.27 to 0.78, with item discrimination ranging from 0.38 to 0.49. The internal consistency coefficients ranged from 0.808 to 0.902. The inter-rater reliability was 0.944, and the intraclass correlation coefficient (ICC) for FMS retest reliability was 0.743. Therefore, the reliability and validity were found to be at a high level, indicating that TGMD-3 can serve as an effective tool for evaluating the development of FMS in Chinese children. Additionally, this study employed the Fundamental Movement Skill Development Test for Children Aged 3 to 10 developed by Diao (2018) to measure children’s stability skills. The difficulty level of the stability skills test ranges from 0.49 to 0.75. The correlation between each movement and the total score of the Stability Skills Test ranges from 0.762 to 0.868. The internal consistency reliability is 0.749. Therefore, it has high reliability and validity. In addition, the tool has also been widely applied in Chinese study (Zhang, 2020). The test indicators for locomotors skills include running, running long jump, standing long jump, hopping on one foot, forward shuffle step, and side shuffle step, totaling six items; the test indicators for manipulative skills include dribbling a ball in place, kicking a ball, catching a ball with both hands, striking a stationary ball, overhand throwing, underhand tossing, and striking a rebounding ball with one hand, totaling seven items; the test indicators for stability skills include standing on one foot, walking on a balance beam, and rolling forward and backward, totaling three items. In total, there are 16 test indicators.

All researchers involved in the testing have been trained and are proficient in imitating each movement and familiar with the process and evaluation criteria for each movement. Prepare the record forms in advance according to the participant list provided by the school, and score each movement based on the standards in the record form. Before the test, check the participants’ attire and the condition of the testing area to minimize the occurrence of dangerous accidents. According to the test requirements, researchers must first demonstrate the correct movements to the participants, who then practice the movements twice before proceeding to the formal test. Each movement has 3–6 evaluation criteria; if a participant meets the evaluation criteria while performing the movement, it is recorded as 1 point, and if the criteria are not met, it is recorded as 0 points. Participants have two opportunities to test the same movement, and the scores from both attempts are added together to determine the final score for that movement. Specifically, running, forward shuffle step, hopping on one foot, standing long jump, and side shuffle step each have 4 evaluation criteria, with a maximum score of 8 points per movement; the cross jump has 3 evaluation criteria, with a maximum score of 6 points. The locomotors skill test score is the sum of the scores from the 6 movements, with a maximum of 46 points. Striking a stationary ball has 5 evaluation criteria, with a maximum score of 10 points; kicking a ball, overhand throwing, underhand tossing, and striking a rebounding ball with one hand each have 4 evaluation criteria, with a maximum score of 8 points per movement; dribbling in place and catching a ball with both hands each have 3 evaluation criteria, with a maximum score of 6 points per movement. The manipulative skill test score is the sum of the scores from the 7 movements, with a maximum of 54 points. Standing on one foot has 6 evaluation criteria, with a maximum score of 12 points; forward and backward rolling has 5 evaluation criteria, with a maximum score of 10 points; walking on a balance beam has 4 evaluation criteria, with a maximum score of 8 points. The stability skill test score is the sum of the scores from the 3 movements, with a maximum of 30 points. The FMS score is the total of the scores from the three test scales, with a maximum of 130 points.

#### 2.4.4 Heart rate

This study utilizes the Polar Team Pro heart rate monitors, which are produced in Finland, to monitor the students’ heart rates, ensuring that each class maintains a moderate intensity level (60–69% of HRmax). In addition, through heart rate monitoring, the exercise load of students in the experimental and control classes was kept at a basically identical level, ensuring the homogeneity between groups. In each class, 10 students are randomly selected to wear the heart rate monitors (5 males and 5 females). The heart rate data is transmitted via cloud to the Huawei MatePad 11.5S tablet.

#### 2.4.5 Demographic information

To ensure that students in the experimental and control classes were basically at the same level, this study conducted a survey on the basic demographic information of students in both classes. The demographic information surveyed in this study includes age, gender, height, weight, and Family Socioeconomic Status (FSES). Age and gender were provided by the head teachers of two classes, while FSES was investigated using the MacArthur Subjective Social Status Scale (Galvan et al., 2023), which surveyed the FSES perceived by the participants’ parents. The specific question was, “In our society, some families are at the top of society, and some families are at the bottom. Overall, where does your family stand in this society?” The options range from 10 points for the highest level to 1 point for the lowest level. This scale has high reliability and validity in Chinese samples and has been widely used by researchers (Chen et al., 2022; Zou et al., 2020).

In addition, this study used a Chinese-made height and weight measuring instrument (model: RGZ-120) to measure the height and weight of children. Children were required to wear light clothing, stand barefoot on the scale, and stand up straight with their chest out and eyes looking forward. Then, the height ruler automatically slides down to the top of the head, and the display shows the height and weight. Researchers recorded the children’s height (cm) and weight (kg), keeping one decimal place. This study obtained the children’s body mass index (BMI) data based on the formula BMI = weight (kg)/height (m^2^).

### 2.5 Mathematical statistics

This study utilized SPSS 25.0 software for data processing and statistical analysis, and GraphPad Prism 8 software for the visualization of results. Continuous variables were described using mean (*M*) and standard deviation (*SD*) for descriptive statistics, while categorical variables were described using frequency and percentage for descriptive statistics. Firstly, the normality of the dependent variable was tested using the one-sample Shapiro–Wilk test in conjunction with P–P plots and Q-Q plots, and it was found that the variable basically conforms to a normal distribution. Therefore, an independent samples *t*-test was used for inter-group comparison analysis. In addition, for gender, this study employed the chi-square (*χ*^2^) test to examine the differences in the number of participants between the experimental group and the control group. Secondly, we used MANOVA to explore the effectiveness of the intervention experiment, with the dependent variables being pre-test and post-test measures of attention, social–emotional skills, and FMS. The independent variable was the teaching program (open-ended physical games vs. closed-ended physical games), and the control variables included heart rate, age, gender, BMI, and FSES. Levene’s test was used for homogeneity of variance; the Pillai trace method was used for model effect testing; and the estimated marginal means (LSD method) were used for pairwise comparisons. Finally, this study employed Pearson partial correlation analysis to explore the correlation between FMS, attention, and social–emotional skills after controlling for related variables following the intervention. All statistical methods mentioned above were set at a significance level of *α* = 0.05.

## 3 Results

### 3.1 Basic information of participants

There were no significant differences in mean age (*t* = −1.013), gender ratio (*χ*^2^ = 0.065), BMI (*t* = −1.027), FSES (*t* = −0.191) and heart rate (*t* = 0.942) between the experimental and control groups (*p* < 0.05), and the demographic information is detailed in Table 1. Therefore, it can be stated that the two groups are homogeneous, which can improve statistical power and enhance the reliability and accuracy of experimental results.

**Table 1:** Basic information of participants.

Variables	Experimental group	Control group	*t/χ^2^*	*p*
Age	7.39 ± 0.50	7.52 ± 0.51	−1.013	0.315
Gender (Girls)	16/51.6%	15/48.4%	0.065	0.799
BMI	19.63 ± 2.53	20.29 ± 2.55	−1.027	0.308
FSES	6.13 ± 1.31	6.19 ± 1.35	−0.191	0.065
Heart rate	131.32 ± 8.44	129.58 ± 5.91	0.942	0.350

### 3.2 Intervention effect of open-ended physical games on attention

**Figure 1:**
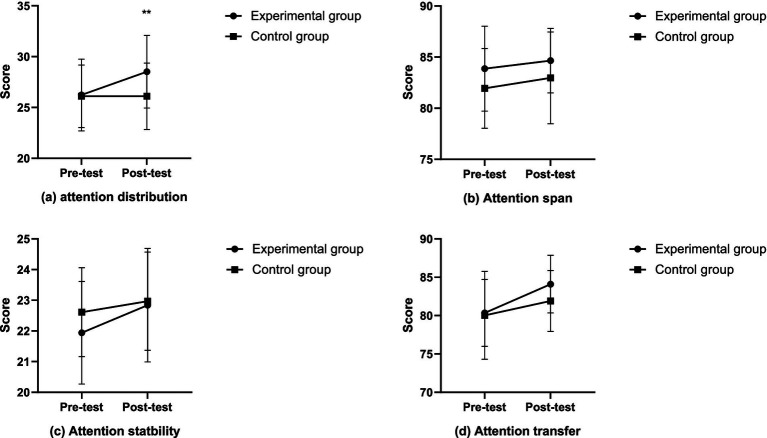
The changes in attention after intervention in the experimental and control groups (*p < 0.05; **p < 0.01).

Firstly, regarding attention distribution, the between-group effect at the pretest was not significant (*F* = 0.045, *p* = 0.833, *η*^2^ = 0.001), while at the posttest, the between-group effect was significant (*F* = 6.223, *p* = 0.022, *η*^2^ = 0.090), with the experimental group (28.52 ± 3.57) showing significantly higher attention allocation than the control group (26.10 ± 3.27). Secondly, for attention span, the between-group effect at the pretest was not significant (*F* = 2.416, *p* = 0.126, *η*^2^ = 0.041), and the between-group effect at the posttest was also not significant (*F* = 2.785, *p* = 0.101, *η*^2^ = 0.047). Thirdly, in terms of attention stability, the between-group effect at the pretest was not significant (*F* = 2.370, *p* = 0.129, *η*^2^ = 0.041), and the between-group effect at the posttest was not significant either (*F* = 1.730, *p* = 0.194, *η*^2^ = 0.030). Lastly, for attention transfer, the between-group effect at the pretest was not significant (*F* = 0.164, *p* = 0.687, *η*^2^ = 0.003), and the between-group effect at the posttest was also not significant (*F* = 3.437, *p* = 0.068, *η*^2^ = 0.058). In summary, the attention qualities of the experimental and control groups were homogeneous before the intervention. However, the attention distribution of the experimental group significantly improved after the intervention. The changes in the experimental and control groups are detailed in Figure 1.

### 3.3 Intervention effect of open-ended physical games on socio-emotional skills

**Figure 2:**
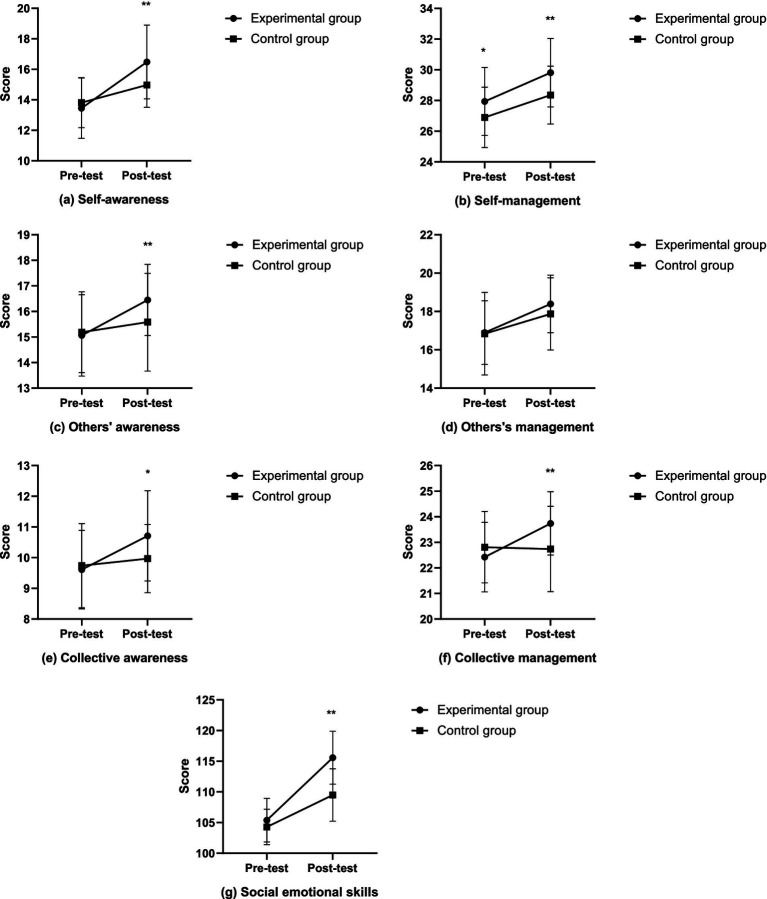
The changes in social emotional skills after intervention in the experimental and control groups (*p < 0.05; **p < 0.01).

Firstly, in terms of self-awareness, the inter-group effect at the pretest is not significant (*F* = 0.493, *p* = 0.485, *η*^2^ = 0.009), while the inter-group effect at the posttest is significant (*F* = 11.027, *p* = 0.002, *η*^2^ = 0.165), with the experimental group (16.48 ± 2.42) showing significantly higher self-awareness than the control group (14.97 ± 1.47). Secondly, in self-management, the inter-group effect at the pretest is significant (*F* = 4.265, *p* = 0.044, *η*^2^ = 0.071), with the experimental group (27.94 ± 2.21) having significantly higher self-management than the control group (26.90 ± 1.96). At the posttest, the inter-group effect is also significant (*F* = 9.203, *p* = 0.004, *η*^2^ = 0.141), with the experimental group (29.81 ± 2.23) maintaining significantly higher self-management than the control group (28.35 ± 1.89). Thirdly, in others’ awareness, the inter-group effect at the pretest is not significant (*F* = 0.021, *p* = 0.885, *η*^2^ < 0.001), while the inter-group effect at the posttest is significant (*F* = 10.315, *p* = 0.002, η^2^ = 0.156), with the experimental group (16.61 ± 1.61) showing significantly higher others’ awareness than the control group (15.58 ± 1.91). Fourthly, in others’ management, the inter-group effect is not significant at either the pretest (*F* = 0.179, *p* = 0.674, *η*^2^ = 0.003) or the posttest (*F* = 1.325, *p* = 0.255, *η*^2^ = 0.023). Fifthly, in collective awareness, the inter-group effect at the pretest is not significant (*F* = 0.001, *p* = 0.982, *η*^2^ < 0.001), while the inter-group effect at the posttest is significant (*F* = 6.494, *p* = 0.014, *η*^2^ = 0.104), with the experimental group (10.91 ± 1.62) having significantly higher collective awareness than the control group (9.97 ± 1.11). Sixthly, in collective management, the inter-group effect at the pretest is marginally significant (*F* = 3.126, *p* = 0.083, *η*^2^ = 0.053), while the inter-group effect at the posttest is significant (*F* = 12.108, *p* = 0.001, *η*^2^ = 0.178), with the experimental group (23.97 ± 1.35) showing significantly higher collective management than the control group (22.61 ± 1.56). Lastly, in overall social emotional skills, the inter-group effect at the pretest is not significant (*F* = 3.339, *p* = 0.073, *η*^2^ = 0.056), while the inter-group effect at the posttest is significant (*F* = 38.453, *p* < 0.001, *η*^2^ = 0.407), with the experimental group (115.58 ± 4.32) showing significantly higher social emotional skills than the control group (109.48 ± 4.27). The changes in social emotional skills after the intervention for both the experimental and control groups are detailed in Figure 2. In summary, after the intervention, the experimental class showed improvements in self-awareness, others’ awareness, collective awareness, collective management, and overall social emotional skills. The changes in social emotional skills after intervention for the experimental and control groups are detailed in Figure 2.

### 3.4 Intervention effect of open-ended physical games on FMS

**Figure 3:**
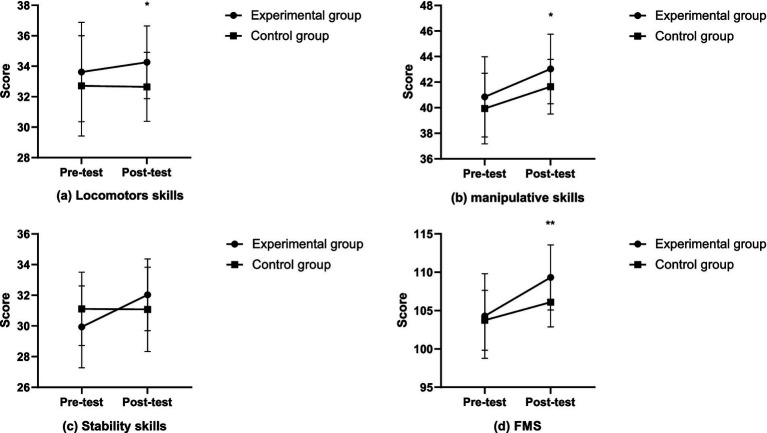
The changes in FMS after intervention in the experimental and control groups (*p < 0.05; **p < 0.01).

Firstly, during the pretest of locomotors skills, there was no significant group effect (*F* = 0.356, *p* = 0.553, *η*^2^ = 0.006). However, during the posttest, the group effect was significant (*F* = 6.458, *p* = 0.014, *η*^2^ = 0.104). After the intervention, the locomotors skills of the experimental group (34.26 ± 2.39) were significantly higher than those of the control group (32.65 ± 2.26). Secondly, during the pretest of manipulative skills, there was no significant group effect (*F* = 1.768, *p* = 0.189, *η*^2^ = 0.031). However, during the posttest, the group effect was significant (*F* = 6.380, *p* = 0.014, *η*^2^ = 0.102). After the intervention, the manipulative skills of the experimental group (43.03 ± 2.73) were significantly higher than those of the control group (41.65 ± 2.14). Thirdly, during the pretest of stability skills, there was no significant group effect (*F* = 3.896, *p* = 0.053, *η*^2^ = 0.065). During the posttest, the group effect was also not significant (*F* = 0.012, *p* = 0.914, *η*^2^ < 0.01). Lastly, during the pretest of FMS, there was no significant group effect (*F* = 0.035, *p* = 0.852, *η*^2^ = 0.001). However, during the posttest, the group effect was significant (*F* = 10.333, *p* = 0.002, *η*^2^ = 0.156). After the test, the overall FMS of the experimental group (109.32 ± 4.24) were significantly higher than those of the control group (106.10 ± 3.22). In summary, the FMS of the experimental group were significantly improved after the intervention. The changes in the experimental and control groups after the intervention are detailed in Figure 3.

### 3.5 Correlation among FMS, attention, and social–emotional skills

#### 3.5.1 Correlation among FMS and attention

The results of the partial correlation analysis between FMS and attention after the intervention (Table 2) show that there is a significant positive correlation between locomotors skills and attention span (*r* = 0.279, *p* < 0.05), and between stability skills and attention distribution (*r* = 0.322, *p* < 0.01). The overall FMS also shows significant (*p* < 0.05) positive correlations with attention distribution (*r* = 0.294) and attention span (*r* = 0.378). However, no association was found between manipulative skills and attention, nor between attention stability and attention transfer and FMS.

**Table 2:** The partial correlation results between FMS and attention after intervention.

Variables	Attention distribution	Attention span	Attention stability	Attention transfer
Locomotors skills	0.062	0.279*	0.005	0.137
Manipulative skills	0.103	0.198	−0.046	0.105
Stability skills	0.322**	0.151	0.057	0.134
FMS	0.294*	0.378**	0.010	0.226

*p < 0.05; **p < 0.01.

#### 3.5.2 Correlation among FMS and social–emotional skills

The results of the partial correlation analysis between FMS and social–emotional skills after the intervention (Table 3) indicate that Locomotors skills have significant (*p* < 0.05) positive correlations with collective awareness (*r* = 0.360) and overall social–emotional skills (*r* = 0.288). Manipulative skills show significant (*p* < 0.05) positive correlations with self-management (*r* = 0.305), collective awareness (*r* = 0.285), and overall social–emotional skills (*r* = 0.368). The overall FMS also exhibits significant (*p* < 0.05) positive correlations with collective awareness (*r* = 0.392), collective management (*r* = 0.269), and overall social–emotional skills (*r* = 0.388). Additionally, no associations were found between stability skills and social–emotional skills, nor between self-awareness, others’ management, collective management, and FMS.

**Table 3:** The partial correlation results between FMS and social–emotional skills after intervention.

Variables	Locomotors skills	Manipulative skills	Stability skills	FMS
Self-awareness	0.090	0.189	0.066	0.207
Self-management	0.195	0.305*	−0.203	0.178
Others’ awareness	0.246	0.148	0.025	0.252
Others’ management	0.088	−0.099	−0.131	−0.086
Collective awareness	0.360**	0.285*	0.006	0.392**
	0.044	0.290*	0.114	0.269*
Social–emotional skills	0.288*	0.368**	−0.012	0.388**

*p < 0.05; **p < 0.01.

#### 3.5.3 Correlation among attention and social–emotional skills

The partial correlation analysis results of attention and social–emotional skills after the intervention (Table 4) show that there is a significant (*p* < 0.01) positive correlation between attention distribution and collective management (*r* = 0.388), and the total social–emotional skills (*r* = 0.359). Attention span is significantly positively correlated with others’ awareness (*r* = 0.349, *p* < 0.01). Attention transfer is significantly positively correlated with others’ awareness (*r* = 0.287, *p* < 0.05). However, no association has been found between attention stability and social–emotional skills, nor has any association been found between self-awareness, others’ management, collective awareness, and attention qualities.

**Table 4:** The partial correlation results between attention and social–emotional skills after intervention.

Variables	Attention distribution	Attention span	Attention stability	Attention transfer
Self-awareness	0.193	0.051	0.057	0.048
Self-management	0.026	0.247	−0.164	0.055
Others’ awareness	0.231	0.349**	−0.012	0.287*
Others’ management	0.036	−0.130	−0.093	−0.096
Collective awareness	0.164	0.002	0.082	0.036
Collective management	0.388**	−0.208	0.009	−0.002
Social–emotional skills	0.359**	0.091	−0.014	0.107

*p < 0.05; **P<0.01.

## 4 Discussion

### 4.1 Open physical games promote attention better than closed physical games

The results of this study indicate that open physical games are superior to closed physical games in promoting children’s attention quality, especially in attention distribution. Additionally, the findings of this study are similar to previous research outcomes (Gökçe et al., 2021; Zhu et al., 2020). For instance, Zhu et al. (2020) quantitatively compared the effects of open and closed skill exercises on visual spatial attention and found that the intervention effects of open skill exercises were more positive. Research on athletes (Gökçe et al., 2021) also found that fencers (open skill athletes) performed better in tasks involving visual spatial working memory and selective attention compared to swimmers (closed skill athletes). The attention quality measured in this study is somewhat related to the visual spatial attention and selective attention examined in previous studies (Gökçe et al., 2021; Zhu et al., 2020). Selective attention involves focusing on certain information while suppressing irrelevant information when faced with numerous stimuli, which is associated with multitasking in attention distribution. Furthermore, visual spatial attention, which involves quickly locating key information in the environment, is inseparable from the roles of attention distribution.

Open physical games take place in an unpredictable environment, which requires children to constantly react and adjust their movements to the changing circumstances. This continuous adaptation and adjustment can enhance the brain’s plasticity, thereby promoting the development of attention (Ball et al., 2019; Biino et al., 2023). In addition, open physical games often involve more interpersonal interactions, and this social element can improve children’s social cognitive levels, including functions such as attention distribution (Bodenhausen and Hugenberg, 2011). Compared to the singular and sustained closed skill exercises, open physical games can promote the generation of positive emotions and the use of emotional regulation strategies, enhancing children’s positive emotional experiences. Shi and Feng (2022) have shown that the aforementioned psychological factors are closely related to attention quality. The combined action of these mechanisms makes open physical games play a significant role in promoting children’s attention development. In addition, Zhou et al. (2020) found that regular open skill exercises can enhance the allocation of attention resources related to perceptual processing, thereby improving cognitive performance. According to the results of this study, physical games with greater cognitive challenges may provide more benefits for certain aspects of attention.

However, this study did not find that open physical games had a positive effect on the other three dimensions of attention quality. This may be related to the learning and practice activities adopted in this study. Simple games based on FMS may not be able to stimulate students’ attention span, transfer, and stability. In addition, the small sample size and the short duration of the intervention may also be potential reasons for the insignificant intervention effects. Based on this, we look forward to future studies exploring complex games based on FMS, increasing the sample size, and extending the intervention period to further investigate the intervention effects of open physical games.

### 4.2 Open physical games promote socio-emotional skills better than closed physical games

The results of this study indicate that open physical games have more positive effects in promoting children’s social–emotional skills, especially in self-awareness, other’s awareness, collective awareness, collective management, and overall social emotional skills. To promote the development of children’s social–emotional skills, it is necessary to provide certain scenarios and activity platforms to enhance their cognitive and comprehension abilities, and to learn to regulate emotions in practice. Social–emotional, as the name suggests, refers to the inner experiences formed when an individual interacts and communicates with others, which can only be manifested in interpersonal relationships (Malti and Noam, 2016). Therefore, the resolution and coping of an individual’s social–emotional dilemmas must also be realized in the interaction of interpersonal relationships. In the process of dealing with emotional dilemmas, it is difficult for an individual to be effective by merely relying on their own regulation of mentality, emotions, and experiences. More dependence is placed on the improvement of environmental conditions and the coordination of interpersonal atmospheres, and on the help others provide when the individual makes efforts to change.

Open physical games typically include games with flexible rules that require creativity and improvisation. These games often do not have fixed rules or can be adjusted according to the participants’ wishes. Open physical games use an open environment to create a context and activity platform where children can fully enjoy freedom. In addition, open physical games are a type of cooperative game that encourages students to engage in teamwork, communication, and problem-solving. Due to the flexibility of the rules, students need to continuously negotiate, adapt, and innovate during the game process, which helps them learn how to cooperate with others, understand the feelings and needs of others, and how to play a leadership role in the team, laying a solid foundation for future integration into the collective. In addition, related studies (Korbel and Paulus, 2018; López-Mondéjar and Pastor, 2017; Portela-Pino et al., 2021) also suggest that teaching practices or programs that provide peer interaction can significantly improve participants’ social–emotional skills and social awareness. Therefore, by changing sports education strategies and actively conducting physical games that are both open and interactive, the development of children’s social and emotional skills can be promoted. Moreover, this study has not yet found that open physical games have a more positive effect on the intervention of other’s management. The reason for this result may be related to factors such as the duration of the intervention in this study. It is hoped that future studies will extend the intervention period to further verify this.

### 4.3 Open physical games promote FMS better than closed physical games

The results of this study indicate that open physical games are more conducive to the improvement of children’s FMS, especially in terms of overall FMS, locomotors skills, and manipulative skills. In fact, many studies (Engel et al., 2021; Wang et al., 2022) have confirmed that FMS intervention programs based on the nature of play can improve children’s FMS, and FMS programs are often dominated by ball games with open attributes. This study is similar to previous research (Dewi and Verawati, 2022; Duncan et al., 2020) on the enhancement of FMS through open activities, for example, Dewi and Verawati (2022) integrated manipulative games into physical education practice and found that manipulative games can stimulate children’s interest in activities, thereby improving their FMS. In addition, Duncan et al. (2020) designed related activities with badminton as the intervention vehicle, and the results showed that children’s FMS were significantly improved after the intervention.

The open physical games created in this study are diverse, including not only basic actions such as dribbling, passing, throwing, and shooting in a stationary state but also interactive manipulative activities involving multiple people in a moving state. These games feature diversity, fun, and entertainment, which are in line with the age characteristics and physical and mental development patterns of children. In addition, China’s new curriculum standards also propose that curriculum content should ensure a foundation, value diversity, focus on integration, and emphasize application, promoting students to form rich sports experiences and fostering the design concept of developing sports abilities. The open physical games designed in this study align with the new curriculum standards, providing a more complex, variable, and challenging environment, including a more diverse range of movement patterns, involving more social interactions, and encouraging children to invest more cognitive resources. This approach stimulates higher levels of student participation and intrinsic motivation, promoting development on multiple levels. Some studies (Alcaraz-Muñoz et al., 2020; Chuang and Kuo, 2016) has confirmed that such activities not only promote the development of children’s sensory integration, emotional regulation, and social interaction skills, but also improve their basic motor skills such as walking, running, jumping, and throwing, as well as the development of large muscle group movements (Dewi and Verawati, 2022; Duncan et al., 2020), and enhance their health-related physical fitness (Jarani et al., 2016). However, this study has not yet found that open-ended sports games have a positive effect on stability skills. Open physical games often take place in open and dynamic environments, where changes in environmental factors can interfere with participants’ mastery of stability skills. Additionally, the rules of open physical games are relatively flexible and may be adjusted continuously according to the actual situation during the game. As a result, participants are unable to form stable behavioral patterns and skill application habits.

### 4.4 Correlations exist between FMS, attention and socio-emotional skills

This study exploratory found that there is a certain degree of correlation between FMS, attention, and social–emotional skills. Firstly, FMS is significantly positively correlated with attention distribution and attention span, which is similar to previous studies exploring the relationship between FMS and children’s cognitive development (Malambo et al., 2022; O'Hagan et al., 2022). Motor skills and cognitive functions share a common neural basis in the brain, especially the prefrontal cortex, which is involved in both motor control and attention control (Faw, 2003). The dorsolateral prefrontal cortex is closely related to the planning, execution, and regulation of movement. It can receive information from other brain regions, such as the sensory cortex, and plan and adjust the goals, directions, and forces of movements (Faw, 2003). It then transmits instructions to downstream brain regions like the motor cortex to precisely control bodily movements. In addition, the prefrontal cortex plays a key role in the allocation, concentration, and maintenance of attention. It can collaborate with other brain regions, such as the parietal cortex, to filter and screen external information, enabling individuals to focus their attention on information relevant to the current task and ignore irrelevant distractions (Faw, 2003). This provides a neurophysiological basis for the connection between FMS and attention. In addition, through the exercise of FMS tasks, children can accumulate experience in multitasking, improve their perception of time and space, and enhance their attentional flexibility (Malambo et al., 2022; O'Hagan et al., 2022). Therefore, the development of FMS is associated with the improvement of attention distribution and attention span.

Secondly, there was a significant positive correlation between FMS and socio- emotional skills, a result similar to the findings of Cheung et al. (2022) with exceptional children, but not consistent with the findings of Salaj and Masnjak (2022) with preschoolers. This may be due to differences in the age and developmental stages of the participants, where children with special needs face more cognitive challenges in social–emotional skills, and thus FMS stimulation can induce changes in their social adaptability. Furthermore, older children, compared to younger children, have the ability to apply FMS in complex social and emotional situations. The reasons for the association between FMS and social–emotional skills are essentially the same as those for the association between FMS and attention, as there is a shared neural basis between the two. Particularly in the prefrontal cortex, there is common neural activation for motor control, decision-making, social skills, and emotional regulation (Faw, 2003; Hiser and Koenigs, 2018). In practice, children gain opportunities to interact with peers through FMS activities and games, which help them learn to resolve conflicts and form friendships. This contributes to improving children’s emotional regulation and self-efficacy (Ma et al., 2024), thereby promoting the development of social–emotional skills.

Finally, there is a significant positive correlation between children’s attention and social–emotional skills. Although discussions about direct evidence of the relationship between the two are relatively scarce, based on the aforementioned discussion of shared neurophysiological mechanisms, there must be some degree of association between them. In addition, Leffert et al. (2000) have explored methods to promote children’s social cognition and enhance children’s social adaptation abilities from the perspective of adjusting attention strategies, which also preliminarily reveals the existence of a connection between attention and social–emotional skills. However, further exploration is needed with more extensive direct evidence regarding the connection between the two.

The significant positive correlation between FMS, attention, and social–emotional skills provides a basis for physical education teachers to design intervention measures or teaching activities. In terms of intervention implementation, teachers can utilize this relationship to design teaching activities, that is, by creating activities that promote the development of FMS, they can also enhance children’s attention and social–emotional skills. However, it should be noted that the correlation between these three factors is of moderate to low degree. This suggests that it is still necessary to design more targeted activities based on the developmental patterns of children’s attention and social–emotional skills in order to promote greater physical and mental health benefits for students.

### 4.5 The value and significance of this study

This study highlights the multiple values of open-ended physical games in children’s development, indicating that interventions during the childhood stage can promote the development of cognitive, emotional, and social skills. The results of this study have positive implications for school educational practices and public health policies. Firstly, school physical education curricula should incorporate more open-ended physical games in their content design to foster better development in students. During the teaching process, teachers can stimulate students’ interest in exercise and promote the improvement of students’ social–emotional skills and attention quality through open-ended physical games that involve teamwork and dynamic situations. Secondly, it is recommended that open-ended physical activities be included in public health policy initiatives to enhance the overall health levels of children. In addition, through open-ended physical activities, mental health issues related to attention deficits and social barriers can be prevented, reducing the need for future psychological therapy. Finally, this study suggests that future guidelines for open-ended physical activities should be developed to encourage children to engage in more such activities, thereby promoting greater physical and mental health benefits for children.

### 4.6 The limitations of this study

Although this study provides valuable insights and results, it also has some limitations. Firstly, the study focuses on children from a specific cultural background and within a certain age group, which limits the generalizability of its conclusions and their adaptability across cultures, as well as their applicability to children of different age groups. Secondly, the relatively short duration of the intervention in this study may lead to insignificant effects on certain indicators. It also limits the observation and assessment of long-term intervention effects. Thirdly, due to the limitations of research conditions, the sample size included in this study is relatively small. Therefore, stratified tests based on gender have not yet been conducted, and it remains unclear whether there are differences in the effects of open physical games on boys and girls. Fourthly, although this study controlled for relevant confounding factors, there may still be potential confounding factors such as family environment that could interfere with the accuracy of the research results. We look forward to future studies controlling for and further exploring these factors. Lastly, the study may reveal more about the correlation between FMS, attention, and social–emotional skills, but it cannot yet determine the causal relationship among the three. This study expects future research to conduct long-term longitudinal experimental studies, increase the sample size, extend the duration of the intervention, examine children from different cultural backgrounds and age groups, control for more potential confounding factors, and explore the dose-effect relationship between open-ended physical game interventions and children’s attention and social–emotional skills. In addition, it is recommended that experiments adopt a multi-point data collection method to reveal the causal relationships among the three.

## 5 Conclusion

This study explores the intervention effects of open-ended physical games based on FMS on children’s attention and social–emotional skills within the context of Chinese culture. The results indicate that open-ended physical games have better intervention effects on FMS, attention, and social–emotional skills compared to closed physical games. Additionally, the study exploratory found that there is a certain degree of positive correlation between children’s FMS, attention, and social–emotional skills. The above findings have positive implications for physical education practice and the formulation of public health policies.

## Data Availability

The raw data supporting the conclusions of this article will be made available by the authors, without undue reservation.

## References

[ref1] Abrahams L. Pancorbo G. Primi R. Santos D. Kyllonen P. John O. P.. (2019). Social-emotional skill assessment in children and adolescents: advances and challenges in personality, clinical, and educational contexts. Psychol. Assess. 31, 460–473. doi: 10.1037/PAS0000591, PMID: 30869960

[ref2] Alcaraz-Muñoz V. Cifo Izquierdo M. I. Gea García G. M. Alonso Roque J. I. Yuste Lucas J. L. (2020). Joy in movement: traditional sporting games and emotional experience in elementary physical education. Front. Psychol. 11:588640. doi: 10.3389/fpsyg.2020.588640, PMID: 33250825 PMC7674785

[ref3] Aly M. Turk-Browne N. B. (2017). How hippocampal memory shapes, and is shaped by, attention. In Hannula D. E. Duff M. C. The hippocampus from cells to systems: Structure, connectivity, and functional contributions to memory and flexible cognition, Berlin, Germany: Springer International Publishing AG, 369–403

[ref4] Ashdown D. M. Bernard M. E. (2012). Can explicit instruction in social and emotional learning skills benefit the social-emotional development, well-being, and academic achievement of young children? Early Childhood Educ. J. 39, 397–405. doi: 10.1007/S10643-011-0481-X

[ref5] Ball N. J. Mercado E. III Orduña I. (2019). Enriched environments as a potential treatment for developmental disorders: a critical assessment. Front. Psychol. 10:466. doi: 10.3389/fpsyg.2019.00466, PMID: 30894830 PMC6414413

[ref6] Barnett L. M. Stodden D. Cohen K. E. Smith J. J. Lubans D. R. Lenoir M.. (2016). Fundamental movement skills: an important focus. J. Teach. Phys. Educ. 35, 219–225. doi: 10.1123/JTPE.2014-0209

[ref7] Biino V. Tinagli V. Borioni F. Pesce C. (2023). Cognitively enriched physical activity may foster motor competence and executive function as early as preschool age: a pilot trial. Phys. Educ. Sport Pedagog. 28, 425–443. doi: 10.1080/17408989.2021.1990249

[ref8] Bodenhausen G. V. Hugenberg K. (2011). “Attention, perception, and social cognition” in Social Cognition (Hove, East Sussex, UK: Psychology Press), 1–22.

[ref9] Cao D. (2016). A comparative study on the positive psychological qualities of college students who exercise and those who do not. Chengdu, china: Chengdu Sport University.

[ref10] Chang Y. K. Pesce C. Chiang Y. T. Kuo C. Y. Fong D. Y. (2015). Antecedent acute cycling exercise affects attention control: an ERP study using attention network test. Front. Hum. Neurosci. 9:156. doi: 10.3389/fnhum.2015.00156, PMID: 25914634 PMC4391039

[ref11] Che X. (2014). A study on the current status and relationship between extracurricular physical exercise and the quality of sports friendship among junior high school students. Suzhou, China: Suzhou University.

[ref12] Chen X. Woo J. Yu R. Chung G. K.-K. Yao W. Yeoh E.-K. (2022). Subjective social status, area deprivation, and gender differences in health among Chinese older people. Int. J. Environ. Res. Public Health 19:9857. doi: 10.3390/ijerph19169857, PMID: 36011511 PMC9408352

[ref13] Chen A.-G. Yan J. Yin H.-C. Pan C.-Y. Chang Y.-K. (2014). Effects of acute aerobic exercise on multiple aspects of executive function in preadolescent children. Psychol. Sport Exerc. 15, 627–636. doi: 10.1016/J.PSYCHSPORT.2014.06.004

[ref14] Cheung W. C. Shen S. Meadan H. (2022). Correlation between motor, socio-emotional skills, and academic performance between young children with and without disabilities. J. Dev. Phys. Disabil. 34, 211–231. doi: 10.1007/s10882-021-09796-8

[ref15] Choi J. W. Han D. H. Kang K. D. Jung H. Y. Renshaw P. F. (2015). Aerobic exercise and attention deficit hyperactivity disorder: brain research. Med. Sci. Sports Exerc. 47, 33–39. doi: 10.1249/MSS.0000000000000373, PMID: 24824770 PMC5504911

[ref16] Chuang T. Y. Kuo M. S. (2016). A motion-sensing game-based therapy to foster the learning of children with sensory integration dysfunction. J. Educ. Technol. Soc. 19, 4–16.

[ref17] Chueh T.-Y. Huang C.-J. Hsieh S.-S. Chen K.-F. Chang Y.-K. Hung T.-M. (2017). Sports training enhances visuo-spatial cognition regardless of open-closed typology. PeerJ 5:e3336. doi: 10.7717/PEERJ.3336, PMID: 28560098 PMC5444361

[ref18] Colomeischi A. A. Duca D. S. Bujor L. Rusu P. P. Grazzani I. Cavioni V. (2022). Impact of a school mental health program on children’s and adolescents’ socio-emotional skills and psychosocial difficulties. Children 9:1661. doi: 10.3390/children9111661, PMID: 36360389 PMC9688343

[ref19] De Greeff J. W. Bosker R. J. Oosterlaan J. Visscher C. Hartman E. (2018). Effects of physical activity on executive functions, attention and academic performance in preadolescent children: a meta-analysis. J. Sci. Med. Sport 21, 501–507. doi: 10.1016/J.JSAMS.2017.09.595, PMID: 29054748

[ref20] De Sousa A. F. M. Medeiros A. R. Rosso S. D. Stults-Kolehmainen M. Boullosa D. A. (2019). The influence of exercise and physical fitness status on attention: a systematic review. Int. Rev. Sport Exerc. Psychol. 12, 202–234. doi: 10.1080/1750984X.2018.1455889, PMID: 39845729

[ref21] Deutsch C. K. Dube W. V. McIlvane W. J. (2008). Attention deficits, attention-deficit hyperactivity disorder, and intellectual disabilities. Dev. Disabil. Res. Rev. 14, 285–292. doi: 10.1002/ddrr.42, PMID: 19072752 PMC3584712

[ref22] Dewi R. Verawati I. (2022). The effect of manipulative games to improve fundamental motor skills in elementary school students. Int. J. Educ. Mathematics Sci. Technol. 10, 24–37. doi: 10.46328/ijemst.2163

[ref23] Diao Y. (2018). Research on the development of basic motor skills in children aged 3 to 10 and educational promotion. Shanghai, China: East China Normal University.

[ref24] Dinas P. C. Koutedakis Y. Flouris A. D. (2011). Effects of exercise and physical activity on depression. Ir. J. Med. Sci. 180, 319–325. doi: 10.1007/s11845-010-0633-9, PMID: 21076975

[ref25] Duncan M. J. Noon M. Lawson C. Hurst J. Eyre E. L. (2020). The effectiveness of a primary school based badminton intervention on children’s fundamental movement skills. Sports 8:11. doi: 10.3390/sports8020011, PMID: 31973023 PMC7076760

[ref26] Engel A. Broderick C. van Doorn N. Hardy L. Ward R. Kwai N.. (2021). Effect of a fundamental motor skills intervention on fundamental motor skill and physical activity in a preschool setting: a cluster randomized controlled trial. Pediatr. Exerc. Sci. 34, 57–66. doi: 10.1123/pes.2021-0021, PMID: 34697254

[ref27] Faw B. (2003). Pre-frontal executive committee for perception, working memory, attention, long-term memory, motor control, and thinking: a tutorial review. Conscious. Cogn. 12, 83–139. doi: 10.1016/S1053-8100(02)00030-2, PMID: 12617864

[ref28] Fisher A. Reilly J. J. Kelly L. A. Montgomery C. Williamson A. Paton J. Y.. (2005). Fundamental movement skills and habitual physical activity in young children. Med. Sci. Sports Exerc. 37, 684–688. doi: 10.1249/01.MSS.0000159138.48107.7D, PMID: 15809570

[ref29] Galvan M. J. Payne B. K. Hannay J. Georgeson A. R. Muscatell K. A. (2023). What does the MacArthur scale of subjective social status measure? Separating economic circumstances and social status to predict health. Ann. Behav. Med. 57, 929–941. doi: 10.1093/abm/kaad054, PMID: 37742041

[ref30] Giglia G. Brighina F. Zangla D. Bianco A. Chiavetta E. Palma A.. (2011). Visuospatial attention lateralization in volleyball players and in rowers. Percept. Mot. Skills 112, 915–925. doi: 10.2466/05.22.27.PMS.112.3.915-925, PMID: 21853778

[ref31] Gökçe E. Güneş E. Arı F. Hayme S. Nalçacı E. (2021). Comparison of the effects of open-and closed-skill exercise on cognition and peripheral proteins: a cross-sectional study. PLoS One 16:e0251907. doi: 10.1371/journal.pone.0251907, PMID: 34086693 PMC8177547

[ref32] Harris K. R. Friedlander B. D. Saddler B. Frizzelle R. Graham S. (2005). Self-monitoring of attention versus self-monitoring of academic performance: effects among students with ADHD in the general education classroom. J. Spec. Educ. 39, 145–157. doi: 10.1177/00224669050390030201

[ref33] Hiser J. Koenigs M. (2018). The multifaceted role of the ventromedial prefrontal cortex in emotion, decision making, social cognition, and psychopathology. Biol. Psychiatry 83, 638–647. doi: 10.1016/j.biopsych.2017.10.030, PMID: 29275839 PMC5862740

[ref34] Ikenouchi-Sugita A. Yoshimura R. Sugita K. Hori H. Yamada K. Sakaue M.. (2013). The effects of a walking intervention on depressive feelings and social adaptation in healthy workers. J. UOEH 35, 1–8. doi: 10.7888/juoeh.35.1, PMID: 23475018

[ref35] Jarani J. Grøntved A. Muca F. Spahi A. Qefalia D. Ushtelenca K.. (2016). Effects of two physical education programmes on health-and skill-related physical fitness of Albanian children. J. Sports Sci. 34, 35–46. doi: 10.1080/02640414.2015.1031161, PMID: 25854535

[ref36] Kalina I. G. Aidarov R. A. Golubev A. I. (2016). Sports activity as a factor contributing to social adaptation of students. J. Org. Cult. Commun. Confl. 20:89.

[ref37] Korbel V. Paulus M. (2018). Do teaching practices impact socio-emotional skills? Educ. Econ. 26, 337–355. doi: 10.1080/09645292.2018.1460320

[ref38] Leffert J. S. Siperstein G. N. Millikan E. (2000). Understanding social adaptation in children with mental retardation: a social-cognitive perspective. Except. Child. 66, 530–545. doi: 10.1177/001440290006600406

[ref39] Li X. Wang X. Dale A. Xu Q. He Y. Guo Q. (2022). A study on the reliability and validity of TGMD-3 in assessing fundamental movement skills among Chinese children aged 3–12 years. J. Wuhan Institute Physical Educ. 56, 86–92. doi: 10.15930/j.cnki.wtxb.2022.03.009

[ref40] López-Mondéjar L. M. Pastor L. M. T. (2017). Development of socio-emotional skills through cooperative learning in a university environment. Procedia Soc. Behav. Sci. 237, 432–437. doi: 10.1016/j.sbspro.2017.02.086

[ref41] Ma X. Yang N. Huang M. Zhan S. Cao H. Jiang S. (2024). Relationships between gross motor skills, psychological resilience, executive function, and emotional regulation among Chinese rural preschoolers: a moderated mediation model. Heliyon 10:e38039. doi: 10.1016/j.heliyon.2024.e38039 39364252 PMC11447319

[ref42] Malambo C. Nová A. Clark C. Musálek M. (2022). Associations between fundamental movement skills, physical fitness, motor competency, physical activity, and executive functions in pre-school age children: a systematic review. Children 9:1059. doi: 10.3390/children9071059, PMID: 35884044 PMC9315971

[ref43] Malti T. Noam G. G. (2016). Social-emotional development: from theory to practice. Eur. J. Dev. Psychol. 13, 652–665. doi: 10.1080/17405629.2016.1196178

[ref44] Merrienboer J. J. V. Schuurman J. G. De Croock M. B. Paas F. (2002). Redirecting learners’ attention during training: effects on cognitive load, transfer test performance and training efficiency. Learn. Instr. 12, 11–37. doi: 10.1016/S0959-4752(01)00020-2

[ref45] Ministry of Education of the People's Republic of China (2022). Compulsory education physical education and health curriculum standards (2022 edition). Beijing: Beijing Normal University Press, 7–9.

[ref46] Ni Q. (2018). The impact of different duration of fancy rope skipping on Children's attention quality and academic performance. Contemp. Sports Technol. 8, 201–202.

[ref47] O'Hagan A. D. Behan S. Peers C. Belton S. O'Connor N. Issartel J. (2022). Do our movement skills impact our cognitive skills? Exploring the relationship between cognitive function and fundamental movement skills in primary school children. J. Sci. Med. Sport 25, 871–877. doi: 10.1016/j.jsams.2022.08.001, PMID: 36064502

[ref48] Petrosini L. De Bartolo P. Foti F. Gelfo F. Cutuli D. Leggio M. G.. (2009). On whether the environmental enrichment may provide cognitive and brain reserves. Brain Res. Rev. 61, 221–239. doi: 10.1016/j.brainresrev.2009.07.002, PMID: 19631687

[ref49] Portela-Pino I. Alvariñas-Villaverde M. Pino-Juste M. (2021). Socio-emotional skills in adolescence. Influence of personal and extracurricular variables. Int. J. Environ. Res. Public Health 18:4811. doi: 10.3390/ijerph18094811, PMID: 33946399 PMC8124598

[ref50] Reloba-Martínez S. Reigal-Garrido R. E. Hernández-Mendo A. Martínez-López E. J. Martín-Tamayo I. Chirosa-Ríos L. J. (2017). Effects of after-school, high-intensity physical activity programme, on levels of attention of school children. Revista Psicologia Deporte 26, 29–36.

[ref51] Salaj S. Masnjak M. (2022). Correlation of motor competence and social-emotional wellbeing in preschool children. Front. Psychol. 13:846520. doi: 10.3389/fpsyg.2022.846520 35465487 PMC9019125

[ref52] Shi J. Cheung A. C. Zhang Q. Tam W. W. Y. (2022). Development and validation of a social emotional skills scale: evidence of its reliability and validity in China. Int. J. Educ. Res. 114, –102007. doi: 10.1016/j.ijer.2022.102007

[ref53] Shi P. Feng X. (2022). Motor skills and cognitive benefits in children and adolescents: relationship, mechanism and perspectives. Front. Psychol. 13:1017825. doi: 10.3389/fpsyg.2022.1017825, PMID: 36478944 PMC9721199

[ref54] Shi P. Tang Y. Zhang Z. Feng X. Li C. (2022). Effect of physical exercise in real-world settings on executive function of typical children and adolescents: a systematic review. Brain Sci. 12:1734. doi: 10.3390/brainsci12121734, PMID: 36552193 PMC9775424

[ref55] Shi P. Zhang Z. Feng X. Li C. Tang Y. (2024). Effect of physical exercise in real-world settings on executive function of atypical children: a systematic review and meta- analysis. Child Care Health Dev. 50:e13182. doi: 10.1111/cch.13182 37873578

[ref56] Sigman M. D. Cohen S. E. Beckwith L. Parmelee A. H. (1986). Infant attention in relation to intellectual abilities in childhood. Dev. Psychol. 22, 788–792. doi: 10.1037/0012-1649.22.6.788

[ref57] Spitzer U. S. Hollmann W. (2013). Experimental observations of the effects of physical exercise on attention, academic and prosocial performance in school settings. Trends Neurosci. Educ. 2, 1–6. doi: 10.1016/J.TINE.2013.03.002

[ref58] Stodden D. F. Goodway J. D. Langendorfer S. J. Roberton M. A. Rudisill M. E. Garcia C.. (2008). A developmental perspective on the role of motor skill competence in physical activity: an emergent relationship. Quest 60, 290–306. doi: 10.1080/00336297.2008.10483582

[ref59] Triviño-Paredes J. Patten A. R. Gil-Mohapel J. Christie B. R. (2016). The effects of hormones and physical exercise on hippocampal structural plasticity. Front. Neuroendocrinol. 41, 23–43. doi: 10.1016/j.yfrne.2016.03.001, PMID: 26989000

[ref60] Tsai C.-L. Wang C.-H. Pan C.-Y. Chen F. Huang S.-Y. Tseng Y.-T. (2016). The effects of different exercise types on visuospatial attention in the elderly. Psychol. Sport Exerc. 26, 130–138. doi: 10.1016/J.PSYCHSPORT.2016.06.013, PMID: 39912375

[ref61] Ulrich D. A. (2013). The test of gross motor development-3 (TGMD-3): administration, scoring, and international norms. Spor Bilimleri Dergisi 24, 27–33.

[ref62] Vanhelst J. Béghin L. Duhamel A. Manios Y. Molnar D. Henauw S. D.. (2016). Physical activity is associated with attention capacity in adolescents. J. Pediatr. 168, 126–131.e2. doi: 10.1016/j.jpeds.2015.09.029 26480921

[ref63] Vhavle S. P. Rao R. M. Manjunath N. (2019). Comparison of yoga versus physical exercise on executive function, attention, and working memory in adolescent schoolchildren: a randomized controlled trial. Int. J. Yoga 12, 172–173. doi: 10.4103/ijoy.IJOY_61_18, PMID: 31143027 PMC6521753

[ref64] Wadlinger H. A. Isaacowitz D. M. (2011). Fixing our focus: training attention to regulate emotion. Personal. Soc. Psychol. Rev. 15, 75–102. doi: 10.1177/1088868310365565, PMID: 20435804 PMC2970710

[ref65] Wang G. Zi Y. Li B. Su S. Sun L. Wang F.. (2022). The effect of physical exercise on fundamental movement skills and physical fitness among preschool children: study protocol for a cluster-randomized controlled trial. Int. J. Environ. Res. Public Health 19:6331. doi: 10.3390/ijerph19106331, PMID: 35627867 PMC9141773

[ref66] Wu P. (2018). A comparative study on the social adaptability of college students engaged in physical exercise and non-exercise. Chengdu, China: Chengdu Sport University.

[ref67] Xiong X. Zhu L.-N. Dong X. Wang W. Yan J. Chen A.-G. (2018). Aerobic exercise intervention alters executive function and white matter integrity in deaf children: a randomized controlled study. Neural Plast. 2018, 1–8. doi: 10.1155/2018/3735208, PMID: 29853843 PMC5952588

[ref68] Yantis S. (2000). Goal-directed and stimulus-driven determinants of attentional control. Control of cognitive processes: Attention and performance XVIII/MIT. Reconfiguration-of-stimulus-task-sets-and-response-task-sets-during-task-switching.pdf

[ref69] Yin H. (2003). Research on the test and evaluation indicators of adolescent attention. China Sport Sci. Technol. Dalian, China. 44, 52–54.

[ref70] Yin S. (2018). The impact of extracurricular physical exercise on the social adaptability of college students: a survey study based on a university. J. Jilin Institute Physical Educ. 34, 48–52.

[ref71] Yin B. (2023). The impact of motor coordination exercises on adolescent attention: Liaoning Normal University.

[ref72] Yu Y. Xu L. Gai R. Cui Y. Yang P. Li J. (2014). Analysis of emotional and behavioral problems and influencing factors among preschool left-behind children in rural Shandong Province. Chin. J. Child Health Care 22, 906–909.

[ref73] Zhang S. (2020). A study on the relationship between basic motor skills and physical activity in preschool children. Beijing, China: Capital University of Physical Education and Sports.

[ref74] Zhao B. Guan M. (2020). Progress in the study of molecular mechanisms of attention deficit hyperactivity disorder in children. J. Baotou Medical College 36, 122–126. doi: 10.16833/j.cnki.jbmc.2020.04.040

[ref75] Zhou Q. (2023). The impact of open-ended physical games on the social-emotional competence of children aged 6–8. Dalian, China: Liaoning Normal University.

[ref76] Zhou M. Wei R. Yuan X. Zhao T. Zhao S. (2023). The intervention effect of group training on the attention quality of preschool children with attention deficit hyperactivity disorder. Chin. J. Child Health Care 31, 336–340.

[ref77] Zhou F. Xi X. Qin C. (2020). Regular open-skill exercise generally enhances attentional resources related to perceptual processing in young males. Front. Psychol. 11:941. doi: 10.3389/fpsyg.2020.00941, PMID: 32508721 PMC7248399

[ref78] Zhu H. Chen A. Guo W. Zhu F. Wang B. (2020). Which type of exercise is more beneficial for cognitive function? A meta-analysis of the effects of open-skill exercise versus closed-skill exercise among children, adults, and elderly populations. Appl. Sci. 10:2737. doi: 10.3390/app10082737

[ref79] Zou H. Xiong Q. Xu H. (2020). Does subjective social status predict self-rated health in Chinese adults and why? Soc. Indic. Res. 152, 443–471. doi: 10.1007/s11205-020-02445-1

